# Variation in Neural Crest Development and Functional Divergence of *sox10* Paralogs Contribute to Morphological Diversification in Cichlid Fishes

**DOI:** 10.1101/2024.01.30.578004

**Published:** 2024-01-31

**Authors:** Aleksandra Marconi, Grégoire Vernaz, Achira Karunaratna, Maxon J. Ngochera, Richard Durbin, M. Emília Santos

**Affiliations:** 1 Department of Zoology, University of Cambridge, United Kingdom; 2 Zoological Institute, University of Basel, Switzerland; 3 Senga Bay Fisheries Research Center, Malawi Fisheries Department, P.O. Box 316, Salima, Malawi; 4 Department of Genetics, University of Cambridge, United Kingdom.

## Abstract

Neural crest (NC) is a vertebrate-specific embryonic progenitor cell population at the basis of important vertebrate features such as the craniofacial skeleton and pigmentation patterns. Despite the wide-ranging variation of NC-derived traits across vertebrates, the contribution of NC to species diversification remains largely unexplored. Here, by leveraging the adaptive diversity of African Great Lakes’ cichlid species, we combined comparative transcriptomics and population genomics to investigate the role of NC development in morphological diversification. Our analysis revealed substantial differences in transcriptional landscapes across somitogenesis, an embryonic period coinciding with NC development and migration. Notably, several NC-related gene expression clusters showed both species-specific divergence in transcriptional landscapes and signatures of positive selection. Specifically, we identified two paralogs of the *sox10* gene as prime NC-related candidates contributing to interspecific morphological variation, which displayed remarkable spatio-temporal expression variation in cichlids. Finally, through CRISPR-KO mutants, we experimentally validated the functional divergence between *sox10* paralogs, with the acquisition of a novel role in cichlid skeletogenesis by *sox10-like*. Our study demonstrates the central role of NC-related processes – in particular those controlled by *sox10*s – in generating morphological diversification among closely-related species and lays the groundwork for further investigations into the mechanisms underpinning vertebrate NC diversification.

## Introduction

The remarkable diversity and complexity of craniofacial structures, pigmentation patterns, and social behaviours within vertebrates is a testament to their outstanding capacity to adapt and exploit a wide range of ecological niches. Much of this phenotypic diversity is intimately connected with the emergence of the neural crest (NC)^1,2^. This embryonic multipotent cell population arises from the dorsal portions of the neural tube and then migrates extensively to finally differentiate into a remarkable range of cell types and tissues, including neurons and glia, pigment cells, craniofacial cartilage and bone, among others^3,4^. These diverse cell lineages later assemble to form complex pigmentation patterns in fish, amphibians and birds, as well as divergent head structures, such as fish jaws, bird beaks or mammalian horns^5–8^.

NC has been primarily studied in the context of its origin, development and function, including developmental disorders involving its derivatives (neurocristopathies)^9^. Studies in model organisms revealed that the gene regulatory networks (GRNs) and developmental processes governing NC specification, migration and differentiation are highly conserved across distantly related species^3,10^. This remarkable evolutionary conservation of the NC programme raises key questions about its evolvability and its potential contribution to the origins of vertebrate diversity. Surprisingly, the role that NC cells play in shaping the microevolution of vertebrate traits remains largely unexplored^1,4,11–13^. This is despite the rapid and extensive diversification of NC-derived structures - a hallmark of vertebrate adaptive radiations of multiple vertebrate clades. Striking examples include the diversification of cranial shapes of *Anolis* lizards, beak morphologies in Darwin’s finches and, perhaps most spectacularly, the craniofacial shapes and colour patterns of cichlid fish radiations in the Great African Rift Lakes^14–17^.

**Fig. 1:**
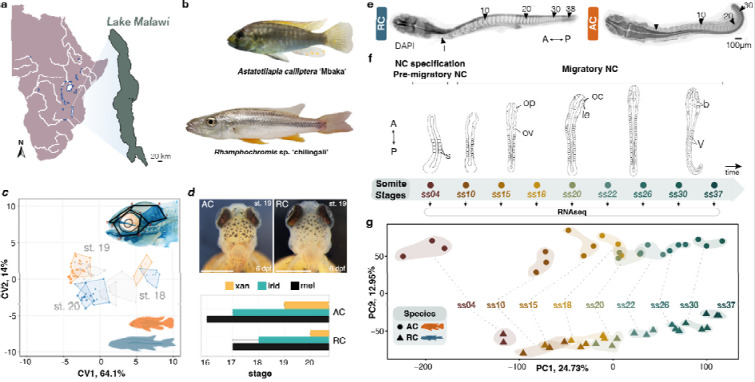
Neural crest-derived phenotypic diversity among Malawi cichlids is established during embryonic development and correlates with divergence in whole-embryo transcriptomic trajectories during neural crest development. **a**, Map of Lake Malawi. **b**, Cichlid species part of this study exhibit distinct neural crest (NC)-derived craniofacial morphologies and pigmentation patterns (to scale). **c, d**, Interspecific divergence in shapes of the craniofacial skeleton (depicted in a common morphospace in **c**) and timing of pigment cell appearance (**d**) during post-hatching development (stages 16–20) indicates that phenotypic variation in these NC-derived traits is specified prior to their overt formation^21^. **e,** AC and RC exhibit different total somite numbers upon completion of somitogenesis. **f,** The stages of cichlid somitogenesis (expressed as somite stages, ss) examined in this study and collected for RNA sequencing range from the early stages of NC specification (4ss) through migratory NC (10–12ss onwards) and its differentiation during late somitogenesis stages, concluding at 30 and 37ss in AC and RC, respectively (n = 3 biological replicates per ss). **f,** Principal Component Analysis (PCA) of whole transcriptome samples reveals significant ontogenic (PC1) and species-specific (PC2) clustering. Each data point corresponds to a pool of three sibling embryos. Figures in (**c-f**) modified from^21^ with authors’ permission. A - anterior, AC - *Astatotilapia calliptera* ‘Mbaka’, dpf - days post-fertilisation, irid - iridophores, le - lens, mel - melanophores, oc - optic cup, op - optic primordium, ov - otic vesicle, P - posterior, RC - *Rhamphochromis* sp. ‘chilingali’, s - somites, ss - somite stage, st - stage, b - tri-partite brain, V - V-shaped somites, xan - xanthophores. Map (**a**) modified from d-maps.com.

Here, we test the extent of variation in NC development and its role in the morphological divergence between two closely related, yet eco-morphologically divergent cichlid species, namely *Astatotilapia calliptera* ‘Mbaka’ and *Rhamphochromis* sp. ‘chilingali’. Both species belong to the Lake Malawi cichlid radiation and are characterised by distinct craniofacial morphologies, body pigmentation, ecologies^3^ and diets^14,18–20^ (Fig. 1a–b). Our previous work revealed quantifiable morphological divergence between these species prior to the overt formation of NC-derived traits, suggesting that variation in pigmentation and craniofacial structures stems from differences in early NC development^21^ (Fig. 1c–d and Extended Data Fig. 1a–b).

To investigate this, we focused on the interspecific comparison at earlier embryonic stages, more specifically segmentation (sequential formation of new somites), concomitant with NC cell specification, migration and onset of their differentiation^22^ (Fig. 1e–f). Given that divergence at the transcriptome level constitutes an important substrate for the evolution of phenotypic variation^23–25^, we first compared whole-transcriptome time-series sequencing data (bulk RNA-seq) and uncovered substantial variation in coding and non-coding gene expression throughout NC development (Fig. 1f–g and Extended Data Fig. 2c–e). This includes divergence in expression levels and temporal trajectories of several genes with key functions within the NC-GRN. Moreover, we show that genes with crucial functions during NC development are also associated with signatures of divergent positive selection, potentially contributing to species-specific differences. We further characterised the embryonic expression patterns of a set of candidate genes, subsequently focusing on two SRY-box transcription factor 10 (*sox10*) paralogs, to elucidate their roles in NC development divergence between the two species. These duplicates – *sox10* and *sox10-like* – arose during fish-specific genome duplication and exhibited substantial spatio-temporal differences within and between cichlid fish species, suggesting both functional divergence and implications for NC migration and fate determination in species divergence. Through a mutagenesis analysis, we provide experimental evidence for a novel role of *sox10-like* in craniofacial skeletal development as well as partial redundancy between *sox10* paralogs, thereby implicating fate divergence of *sox10* duplicates during phenotypic diversification of NC-derived traits in cichlid fishes and teleosts more broadly.

Taken together, our study uncovers a novel role for *sox10-like* in skeletogenesis and identifies extensive variation and divergent selection in NC developmental programmes between two closely related cichlid species. The emergence of such novel craniofacial skeletal function for *sox10-like,* possibly unique to cichlids and divergent from fates of *sox10* duplicates in medaka, demonstrates that NC gene regulatory networks are extremely labile both at the micro- and macro-evolutionary scales.

## Results

### Genes involved in NC development show species-specific shifts in transcriptomic trajectories

Our previous work has shown that phenotypic divergence between cichlid species in NC-derived craniofacial skeleton and body pigmentation is already established at the first appearance of cartilage and pigment cells, respectively^21^ (Fig. 1c–d). Thus, we hypothesise that variation in processes of NC development occurring earlier in embryogenesis might constitute an important source of morphological divergence between these species.

To first investigate the potential role of gene expression variation in driving divergent NC-derived phenotypes, we performed transcriptome profiling (RNAseq) of whole embryos of *Astatotilapia* and *Rhamphochromis* across somitogenesis. This period of embryonic development coincides temporally with NC development^22^ (Fig. 1e). In total, 32.49 ± 2.5 Mio paired-end 150bp-long reads were generated for each sample (three biological replicates per ss), and then aligned against the *Astatotilapia calliptera* reference genome to quantify gene expression (Supplementary Table 1 and Methods). Principal component analysis (PCA) of protein-coding transcriptomes revealed that gene expression in these species is primarily dictated by ontogeny (i.e., their developmental age in somite stage, ss; PC1, 24.73%), followed by species (PC2, 12.95%; Fig. 1g and Extended Data Fig. 1c). Conversely, the expression of non-coding transcripts and transcribed transposable elements (TEs) is primarily clustered by species and then by ontogeny (Extended Data Fig. 1d–f), confirming higher evolutionary rates^24^.

**Fig. 2:**
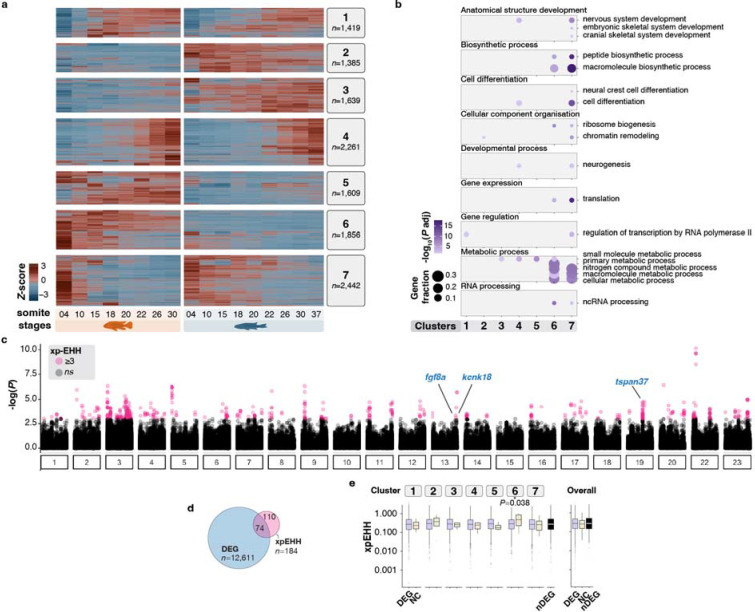
Comparative characterisation of cichlid transcriptomes during somitogenesis and neural crest development. **a,** Heatmap of all differentially expressed genes (DEGs, n=12,611) between any pairwise somite stage comparisons between species identifies seven clusters of gene expression patterns**. b**, Distinct gene ontology categories are significantly enriched in each of the seven clusters of gene expression identified. **c**. Genome-wide scans between *Astatotilapia* and *Rhamphochromis* populations reveal 551 significant SNPs (in pink) showing elevated local haplotype homozygosity (measured using between-population extended haplotype homozygosity, xpEHH; see also Ext. Data. Fig. 2b). **d.** Significant SNPs in close proximity (Methods) were grouped into 154 islands, which were associated with 74 DE genes. **e**. NC-DEGs within cluster 6 show significantly elevated xpEHH values, in line with signatures of positive selection. DEG, differentially expressed genes; nDEG, genes lacking significant expression differences; NC-DEG, DE genes linked to NC development.

We then performed differential gene expression analysis across somitogenesis stages to identify gene candidates showing species- and time-specific transcriptional patterns. In total, 12,611 differentially expressed genes (DEGs; p<0.05) with >1.5-fold expression difference in at least one pairwise comparison were identified (Fig. 2a, Extended Data Tables 1–2 and Methods). Of these, 14.7% (n=1,857) lacked assigned names in the current assembly *A. calliptera* genome (Ensembl 108), likely representing genes without zebrafish orthologs or novel genes and were excluded from downstream analyses. The remaining DEGs were then classified into seven distinct clusters based on unbiased grouping according to their expression patterns (Fig. 2a and Extended Data Fig. 1g). While four of these clusters displayed consistent high or low gene expression in one species across all somite stages (clusters 2, 3, 5 and 6; accounting for 51.4% of all DEGs), the other clusters (1, 4 and 7) showed species-specific temporal shifts in gene expression (Extended Data Fig. 1g). Each gene expression cluster was significantly enriched for specific Gene Ontology (GO) categories associated with functions ranging from transcription regulation to metabolic and developmental processes (Fig. 2b). Notably, clusters 4 and 7, displaying temporal shifts in gene expression dynamics between the two species during early and late somitogenesis, were significantly enriched for genes with functions related to neural crest cell differentiation (Fig. 1a–b and Extended Data Fig. 1g). Altogether, the considerable divergence in expression dynamics between species over developmental time (e.g., temporal shifts) implies variation in multiple developmental processes during embryogenesis, including those involving NC cells. Combined with the diverse repertoire of NC derivatives, these results implicate the potential role of this cell population in interspecific divergence at a microevolutionary scale.

### Signatures of positive selection are associated with NC-related genes

To further explore the link between species divergence and variation in the transcriptomic and genetic landscapes, we conducted genome-wide scans for regions under positive selection. Through extended haplotype homozygosity (xp-EHH^26^) between *Astatotilapia* and *Rhamphochromis* populations (n=43–45 genomes per population; Extended Data Fig. 2a, Extended Data Table 3 and Methods), we identified 154 regions potentially under positive selection, each comprising 1 to 39 Single Nucleotide Polymorphisms (SNPs) (Fig. 2c). Altogether, 74 DEGs were located near or within these putative islands of selection (Fig. 2d, Extended Data Fig. 2b–c; see Methods), having functions related to cell differentiation, signal transduction, metabolic pathways, and several unannotated novel genes (Extended Data Fig. 2e).

Among the annotated DEGs located in the most extreme outlier regions were *fgf8a* (cluster 7), *kcnk18* (cluster 1) and *tspan37* (cluster 5), which exhibited significant expression differences between the two species during the early and mid-phases of somitogenesis (Extended Data Fig. 2 b–c). tspan37 is an integral membrane protein involved in cellular signalling, while *kcnk18* encodes a potassium channel expressed in the brain and eye of hatchling and larval zebrafish^27^. To test whether these genes were involved in NC development and characterise their expression patterns, we performed *in situ* Hybridisation Chain Reaction (HCR)^28^. Expression of *kcnk18* was not detected in whole mount embryos at the stages of differential expression, likely due to overall low expression levels, whereas *tspan37* was not investigated further due to its unlikely role in NC development. Furthermore, *fgf8a*, known for its diverse roles during embryogenesis including in chondrogenesis of the NC-derived cranial skeleton^29^ showed comparable expression only in the developing brain and posterior-most notochord during somitogenesis in the examined cichlids (Extended Data Fig. 3), consistent with findings in zebrafish^30^. This limited expression pattern suggests an unlikely role in craniofacial development and patterning at this stage. Altogether, the most extreme DEG outliers are unlikely to play a role in NC development divergence between these two cichlid species. However, NC genes belonging to cluster 6 (identified based on their GO annotation, Extended Data Table 1, Methods) showed significant enrichment for sites under potential positive selection (Fig. 2e and Extended Data Fig. 2d). These included, among others, the transcription factor *pax3a*, already implicated in cichlid interspecific pigment pattern variation^31^, and the cellular nucleic acid-binding *cnbpa,* involved in craniofacial development in fish^32^. Collectively, these findings highlight a strong association between sequence and transcriptional differences between the two species, with, notably, several DEGs with functions related to NC pathways showing enrichment for sites under potential positive selection.

### Variation in the NC gene regulatory network is pervasive across all developmental modules and is associated with NCC migration

**Fig. 3:**
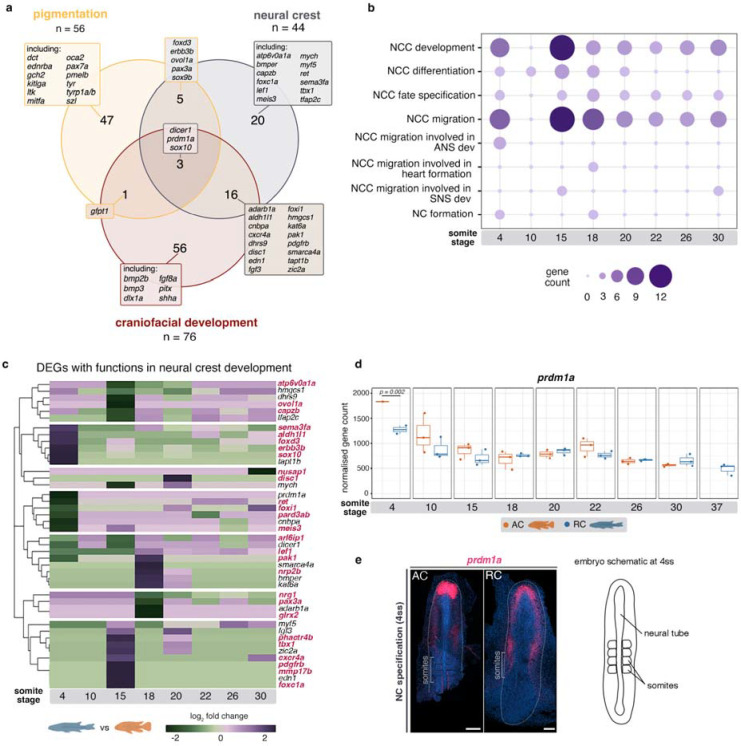
**a,** Overview of DEGs from the NC-GRN, highlighting genes with known roles in development of NC-derived pigmentation and craniofacial skeleton. **b,** distribution of identified DEGs with roles in different tiers of NC developmental programme, from specification to migration and differentiation. **c,** fold changes in expression levels of candidate genes involved in NC development between cichlids. Genes involved in NCC migration highlighted in red. **d,** expression profiles of *prdm1a* during NC development. **e,** *in situ* HCR image showing *prdm1a* expression at 4ss, representative of n ≥ 2 per species. Anterior to the top of the figure. AC - *Astatotilapia calliptera* ‘Mbaka’, ANS - autonomic nervous system, NCC - neural crest cells, RC - *Rhamphochromis* sp. ‘chilingali’, SNS - sympathetic nervous system, ss - somite stage. Scale bar = 100 μm.

Since our comparative transcriptional and selection analyses implicated divergence in NC development between species, we next sought to specifically explore which genes, processes and stages of NC ontogeny could be involved in the generation of morphological variation. To this end, we examined only DEGs with known functions (based on their GO annotation, Extended Data Table 1) in the development of the NC and its two hyper-diverse derivatives, namely pigmentation and craniofacial skeleton (Fig. 3a)

The identified NC DEGs represented various tiers of the NC-GRN^10,33,34^. These included genes involved in NC induction (*bmp* and *fgf* family genes), NC specification (*foxd3, sox9b* and *sox10*), NC migration (e.g. *sox10, sema3fa*) and NC differentiation (Fig. 3a–b). This last category included numerous genes associated with the development of different pigment cell lineages, such as melanophores (*dct, tyr, tyrp1a/b, pmelb, oca2*), xanthophores (*pax3a* and *gch2*) and iridophores (*foxd3, ltk*)^35^ (Fig. 3a). Multiple genes contributing to the development of the embryonic cranial skeleton, such as *dlx1a, fgf8a, pitx2 and shha,* also showed divergence in expression between species*.* Moreover, four signalling pathways - Bmp, Fgf, Hedgehog and Wnt - were implicated by differential expression of *bmper, fgf8a, shha* and *szl,* respectively. Almost 40% of the identified NC-DEGs (Fig. 3a) performed functions in NCC migration (GO:0001755), and most were differentially expressed at 4ss and 15ss (Fig. 3b). These genes (highlighted in red in Fig. 3c) exhibited variation in expression levels over time, often displaying large differences in relative expression between species at individual stages, such as 4, 15 and 18ss (Fig. 3c). These findings might reflect divergence in gene activity but also indicate variation in the sizes of cell populations expressing each of these genes. Furthermore, the considerable number of DEGs involved in NC migration and differentiation might result from broad effects of divergence at the higher tiers (i.e. during NC specification) within the NC-GRN.

Three genes - *sox10, prdm1a* and *dicer1 -* were located at the intersection of higher (specification and migration) and lower NC-GRN tiers (pigmentation and craniofacial skeletal differentiation). *sox10* is a key regulator of NC specification, maintenance, migration and differentiation into multiple cell lineages, primarily neuronal and pigment cells, across vertebrates^36–38^. *prdm1a* controls NC cell formation by activating *foxd3* (an early NC specifier gene) and regulating *sox10* in zebrafish^39–41^. *dicer1* is required for craniofacial and pigment cell development, and together with miRNAs, it is involved in the regulation of *sox10* during melanophore differentiation in zebrafish^42^.

In light of the considerable number of genes involved in NCC migration and differences in expression levels of *sox10* (a regulator of NCC migration), *prdm1a* and *dicer1* (factors regulating *sox10*), we examined the expression patterns of these genes during stages of differential expression in cichlid embryos. Both *prdm1a* and *dicer1* were differentially expressed at 4ss, a stage concurrent with NC specification (Extended Data Fig. 2c). Notably, *dicer1* was not detected in whole-mount specimens. At 4ss in *Astatotilapia, prdm1a* was highly expressed in the prechordal plate (anterior-most tip of the neural tube) and at lower levels along the neural tube, whereas in *Rhamphochromis*, it was expressed at lower and uniform levels in the prechordal plate on both sides of the anterior neural tube (Fig. 3c–d). Considering the positive regulatory role of *prdm1a* on *sox10*^41^, interspecific expression differences in early NC ontogeny will influence later behaviour of migratory NCCs and their differentiation in lineages regulated by *sox10*, such as pigment cells and cartilage. Given this and the elevated numbers of DEGs involved in NCC migration, we next focused on characterisation of this process in cichlid embryos using *sox10* expression as a pan-marker of migratory NCCs.

### Differences in expression between *sox10* paralogs and between cichlid species indicates divergence in NC migration and differentiation

**Fig. 4:**
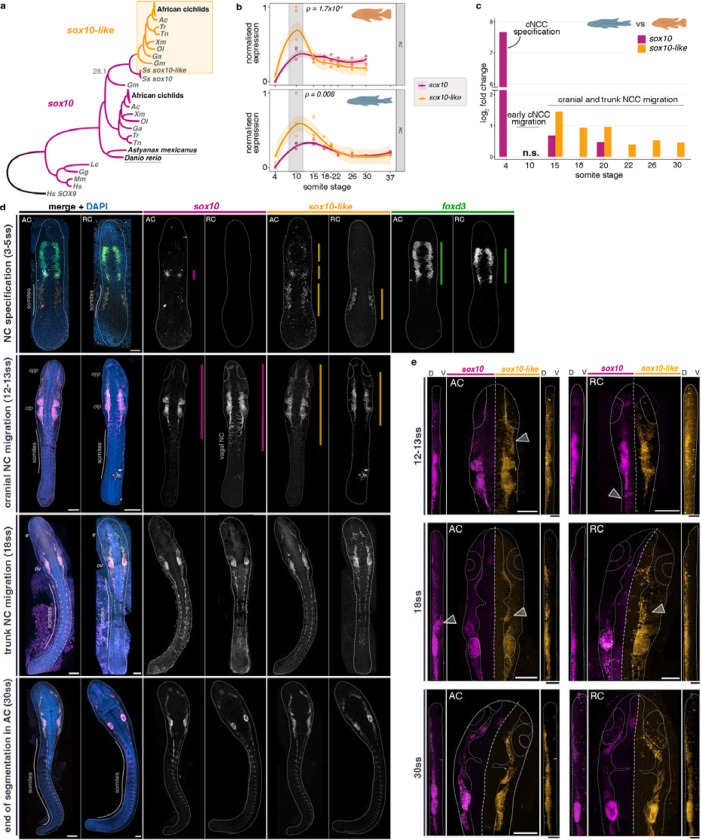
Gene- and species-specific variation in embryonic expression of *sox10* paralogs during neural crest development in Malawi cichlids. **a,** Maximum Likelihood phylogeny of *sox10* and *sox10-like* coding sequences across vertebrates. Underlined species are teleosts with a single *sox10* gene. All significant Bootstrap values, apart from the ones shown (<75). **b,** The expression trajectories of *sox10* paralogs across NC development in *Astatotilapia calliptera* ‘Mbaka’ (AC) and *Rhamphochromis* sp. ‘chillingali’ (RC). Shaded bands indicate 95% confidence intervals. Significant differences in normalised gene counts between *sox10* paralogs were observed at 10ss in both examined species (two-way ANOVA and Tukey HSD, *p* < 0.01). **c,** Fold changes in expression levels of *sox10* and *sox10-like*. Only significant comparisons are shown (*P*-adj < 0.05). **d,** *in situ* HCR images of *foxd3, sox10* and *sox10-like* in representative somite stage-matched embryos of AC and RC. **e,** Side-by-side comparison of *sox10* paralog expression in the cichlid embryonic head showing differences in migratory patterns of NCCs. All images present maximum intensity projections of dorsal or lateral (vertical panels in **e**) views of dissected embryos, with anterior towards the top of the figure. Embryos and sections are outlined in white. Colour-coded vertical bars represent the AP extent of expression of each presented gene. Embryos presented in d and e representative of n ≥ 3. Species acronyms in (a): *Ac* - Midas cichlid *Amphilophus citrinellus*, *Ga* - stickleback *Gasterosteus aculeatus, Gg* - chicken *Gallus gallus*, *Gm* - cod *Gadus morhua*, *Hs* - human *Homo sapiens*, *Lc* - coelacanth *Latimeria chalumnae*, *Mm* - mouse *Mus musculus*, *Ol* - medaka *Oryzias latipes*, *On* - tilapia *Oreochromis niloticus*, *Ss* - salmon *Salmo salar*, *Tn* - tetraodon *Tetraodon nigroviridis*, *Tr* - fugu *Takifugu rubripes*, *Xm* - platyfish *Xiphophorus maculatus*. AC - *Astatotilapia calliptera* ‘Mbaka’, AP- anterior-posterior, D - dorsal, NC - neural crest, n.s. - not significant, RC - *Rhamphochromis* sp. ‘chilingali’, ss - somite stage, V - ventral. Scale bar = 100 μm.

Like other soxE gene family members, *sox10* was duplicated during teleost whole-genome duplication, resulting in paralogs found in cichlid and other teleost genomes^43^ (Fig. 4a). However, unlike some other lineages, such as the teleost model zebrafish, where *sox10* duplication was not retained^43,44^, the functional roles of *sox10* paralogs in NC development remain underexplored. Cichlids are thus an exceptional system to investigate the impact of *sox10* duplication on the evolution of NC and morphological diversification across multiple closely related teleost species.

Our transcriptomic profiling revealed that expression of *sox10* duplicates, referred to here as *sox10* and *sox10-like* (corresponding to *sox10b and sox10a* of other teleosts*,* respectively) followed similar trajectories over time in both species, with a significant difference in transcript levels between paralogs observed at 10ss (two-way ANOVA and Tukey HSD, p<0.001) (Fig. 3b). Further, the *sox10* paralogs were differentially expressed between species across multiple stages of NC development, with differences in *sox10* levels observed at earlier stages compared to *sox10-like* (Fig. 3b). Finally, the fold expression differences for both genes decreased with developmental time (Fig. 3c).

Variation in expression between paralogs within an embryo, was first identified during cranial NC specification (4ss) and manifested in temporal and spatial aspects common to both examined species. First, *sox10-like* was expressed earlier than *sox10* (4ss in Fig. 4a, Extended Data Fig. 4b). Second, *sox10-like* was detected in cells residing bilaterally on the dorsal surface of somites and that did not express neither *sox10* nor the canonical early NCC marker *foxd3* (Fig. 4d, Extended Data Fig. 4b). These distinct expression domains of *sox10-like* compared to its paralog *sox10*, also unreported for any other *soxE* family gene in other teleost species to date^45–47^, suggest that *sox10-like* could have been co-opted to function in non-NC cells in early somitogenesis in cichlids. This hypothesis was further supported by the presence of *sox10-like*+ cells in the extra-embryonic tissues in both species at different stages of development (Extended Data Fig. 4d). Taken together, these findings suggest possible neo-functionalisation of *sox10-like* and warrant further investigation into the embryonic origins and function of this novel cell population.

As the development progressed, the variation in expression levels and spatial arrangement of *sox10* and *sox10-like* domains visibly decreased, resulting in largely overlapping patterns during cranial and trunk NC migration (18ss in Fig. 4d). Nonetheless, we observed considerable fine-scale variation in the cellular behaviour of cranial and trunk NCCs (as labelled by one or both paralogs) between cichlid species. The most pronounced differences involved the anterior-posterior (AP) limits of *sox10* and *sox10-like* expression in stage-matched embryos (vertical coloured bars in Fig. 4d), linked to both the progression of NC specification along the AP axis and the extents of cranial NC migration into and around head structures (e.g. otic placodes/vesicles, optic primordia/eyes and pharyngeal arches) (Fig. 4e). For instance, at 12–13ss, migration of *sox10-like*+ cells between otic and optic placodes extended further laterally (away from midline) and anteriorly (towards the nascent eye) in *Astatotilapia* compared to *Rhamphochromis* (grey arrowhead in Fig. 4e). At the same stage, *sox10*-expressing cells were limited to areas surrounding otic placode in both species but again varied in the extents of lateral migration between species. The fine-scale variation in migratory patterns of *sox10* and *sox10-like*+ NCC populations persisted during cranial migration until the end of somitogenesis (Fig. 4e, Extended Data Fig. 4e). Such spatial differences could be related to fine-scale patterning of the structures derived from cranial NCCs i.e. craniofacial cartilages and bones, connective tissues and pigment cells^48–50^. Combined with differences in expression levels (Fig. 4a–b), these differences could be also related to size variation of the populations expressing each paralog, especially considering the differences in embryo sizes between these species^21^.

Taken together, our results reveal that *sox10* paralogs are expressed in both overlapping and gene-specific domains in cichlids during NC development, implicating both potential functional redundancy and partitioning between *sox10* and *sox10-like* functions in this clade. Finally, the variation between species in expression patterns and, thus, NCC behaviour, strongly suggests a key role in formation of divergent NC-derived phenotypes.

### Genome editing reveals functional divergence between *sox10* paralogs and a novel craniofacial skeletal function for *sox10-like* in cichlid fishes

**Fig. 5:**
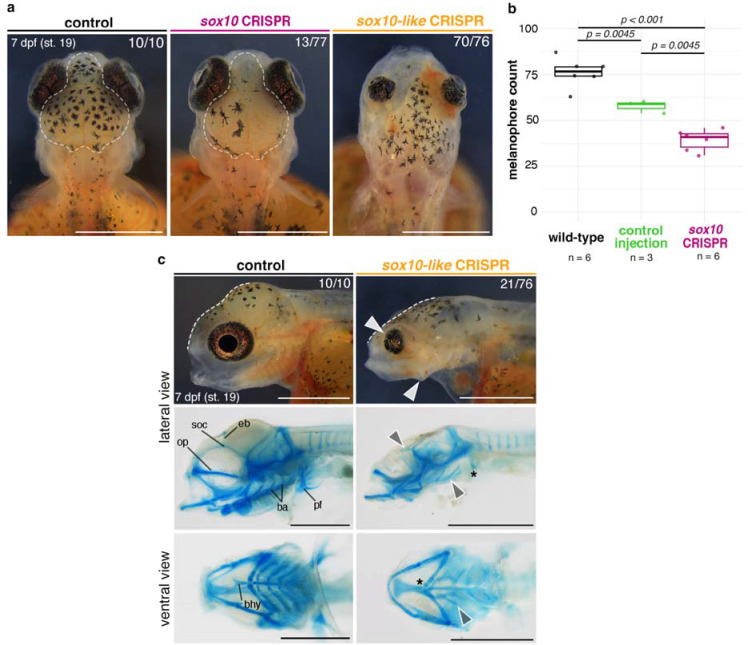
Paralog-specific knockout phenotypes indicate functional divergence of *sox10* and *sox10-like* in cichlids. **a**, Melanophore pigmentation defects are observed in *sox10* but not in *sox10-like A. calliptera* mutants. **b,** *sox10*-CRISPR embryos have significantly reduced melanophore counts at 7 dpf compared to WT and control injections (boxplots showing melanophore count within the dorsal head area outlined in a.; *P*-values are shown for Tukey HSD). **c,** Representative craniofacial phenotypes of *sox10-like-*CRISPR embryos in live animals and their corresponding cartilage preparations in lateral and ventral views. White dashed lines show the reduced frontal slope of the brain case. Arrowheads and asterisks in panels showing cartilage preparations indicate reduced and missing cartilages, respectively, in the mutants. Fractions indicate the frequency of presented phenotype across all surviving embryos on that day. Data shown are representative of at least three biological replicates per target gene. Ba - branchial arches, bhy - basihyal, op - orbital process, soc - super-orbital cartilages, pf - pectoral fin, WT - wild-type. Scale bars = 1 mm.

The expression of *sox10* paralogs in both overlapping and distinct domains during NC development in cichlids could be related to divergence in developmental functions between two genes. Such a functional divergence has not been shown before given that *sox10* paralog function remains uncharacterised beyond the zebrafish (*Danio rerio*) and medaka (*Oryzias latipes*) model systems. In zebrafish, *sox10* function is limited to pigmentation and neural derivatives^38^, while in medaka both paralogs have redundant functions in pigmentation development^46^. To test whether *sox10* and *sox10-like* perform divergent roles in cichlids, we used CRISPR/Cas9 system in *Astatotilapia* to induce indel mutations in the coding sequence (exon 1) of *sox10* and *sox10-like* in turn (Fig. 5, Extended Data Fig. 5–6).

From day 6–7 post-injection (st. 18–19), coinciding with the main stages of craniofacial cartilage development and patterning in this species^21^, craniofacial malformations were observed in *sox10-like* CRISPR mosaic embryos. Neurocranial and craniofacial deformities were prevalent among injected embryos (Fig. 5a, n = 21/76 across four clutches, Supplementary Table 4) and, while ranging in severity between clutch-mates, these mutants consistently exhibited flattening of the frontal bones (brain case) (indicated by white dashed lines in Fig. 5a), small and bulging, forward-facing eyes as well as protruding, unmoving jaws (white arrowheads in Fig. 5a). Alcian Blue stains for cartilage further revealed severely malformed or entirely missing super-orbital cartilages, basihyal, branchial arches and pectoral fins (grey arrowheads and asterisks in Fig. 5a). Besides craniofacial abnormalities, *sox10-like* mutants at this stage also displayed cardiac and circulatory system defects, reduced black melanophore pigmentation and malformed caudal fin cartilages (Extended Data Fig. 5). Embryos with severe *sox10-like*-KO phenotypes (Fig. 5c and Extended Data Fig. 5) did not survive past 9 dpf (st. 22), suggesting embryonic lethality of the KO.

Unlike *sox10*-like CRISPR mosaic embryos, *sox10* crispants did not show any craniofacial cartilage defects at day 7 post-injection (st. 19), but instead had reduced melanophore pigmentation on the dorsal head region (Fig. 5b–c, n = 13/77 across three clutches, Supplementary Table 4), the first body area populated by all three pigment cell types^21^. The development of other pigment cell lineages (i.e., reflective iridophores and yellow xanthophores) appeared unaffected by the induced mutations, with no noticeable differences in development of the flank pigmentation at 12 dpf (st. 24) (Extended Data Fig. 6).

Taken together, these functional analyses provide compelling evidence for divergent roles of cichlid *sox10* and *sox10-like* in the development of the neural crest and its derivatives, pigment cells and cartilage, respectively. Although we observed a level of functional redundancy between paralogs in the differentiation of pigment lineages, we uncovered a pivotal role for *sox10-like* in the formation of cranial skeleton (neurocranium and craniofacial cartilages) - a novel function only described in cichlids.

## Discussion

**Fig. 6:**
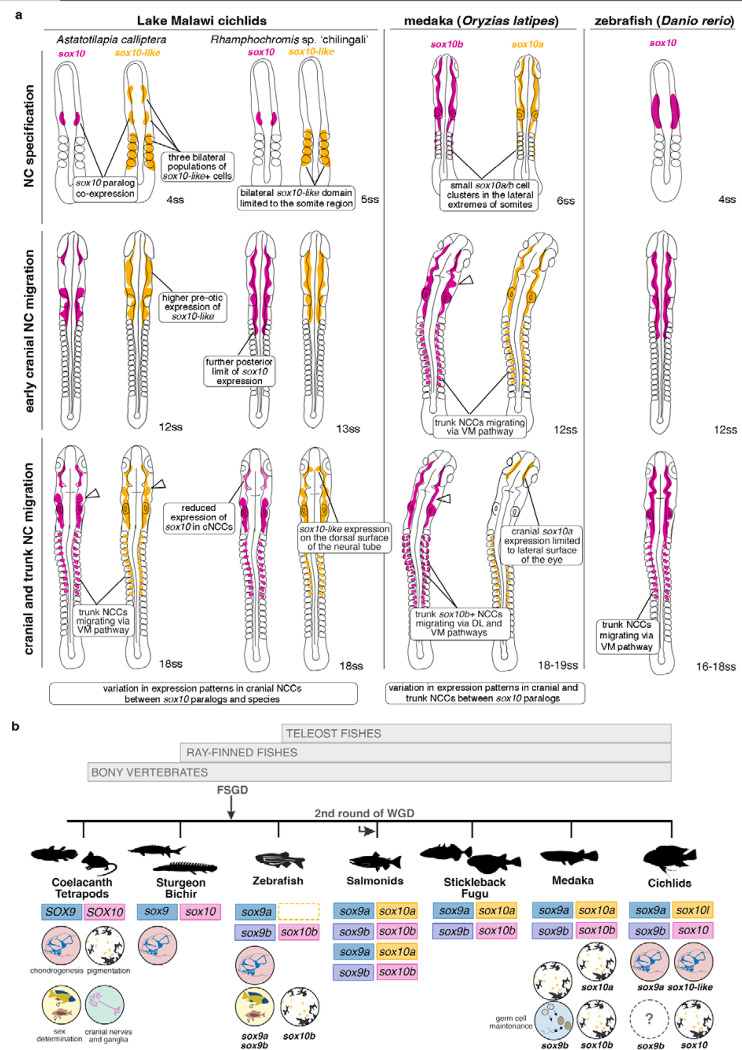
Summary of *sox10* paralog expression data in Malawi cichlids, medaka and zebrafish and functions of *sox10* and *sox9* across the bony fish phylogeny. **a,** Schematic representations of *sox10* paralog expression patterns in Lake Malawi cichlids (this study) and model teleosts medaka (*Oryzias latipes*) and zebrafish (*Danio rerio*) at three representative stages of NC development - NC specification, cranial and trunk NCC migration. Note that medaka at 6 and 12ss are developmentally more advanced (i.e., in formation of eye and otic vesicles) compared to stage-matched cichlids and zebrafish embryos. **b,** Evolution and functions of *sox9* and *sox10* genes across bony fishes phylogeny. Dashed line box indicates gene loss. Bony fish lineage adapted from Ref.^50^. Expression patterns for medaka in (a) based on Ref.^45,46^, expression data for zebrafish based on Ref.^29,46^. References for functional analyses are presented in Extended Data Table 3. Somite stages (ss) for zebrafish and medaka given as ranges when not specified by source material. DL - dorsal-lateral, FSGD - fish-specific genome duplication; NC - neural crest, NCC - neural crest cell; *sox10l* - *sox10-like*, ss - somite stage, VM - ventral-medial, WGD - whole genome duplication. Silhouettes downloaded from http://phylopic.org.

Neural crest development is remarkably conserved across vertebrates, despite NC-derived structures constituting some of the most diverse phenotypic traits, especially among lineages that have undergone adaptive radiation. However, the extent to which NC development evolution contributes to phenotypic diversification remains largely unexplored^1,2,4^. Here, we addressed this paradox by leveraging the recent explosive radiation of African Great Lakes’ cichlid species, renowned for their extreme phenotypic diversity. Our study focused on the transcriptomic and developmental underpinnings of morphological divergence in NC-derived traits, such as craniofacial skeleton and pigmentation. To this end, we employed an integrative approach, combining extensive comparative transcriptomics and population genomics between two eco-morphologically distinct yet closely-related cichlid species across somitogenesis – a developmental period coincident with NC specification, development and migration. Our analysis revealed substantial differences in transcriptomic landscapes and expression patterns between the two species during NC development (Fig. 6), implying contribution to interspecific NC-derived morphological variation among cichlids. Through CRISPR/Cas9 genome editing in *Astatotilapia*, we showed that *sox10-like* knockout mutant is associated with aberrant craniofacial skeletal development phenotypes, consistent with the emergence of functional divergence between *sox10* paralogs in cichlid fishes. These findings suggest that NC development-associated transcriptional evolution contributes to morphological diversification in closely-related species and, more broadly, plays a pivotal role in phenotypic diversification across vertebrates.

Recent studies have highlighted that transcriptional evolution among closely-related species and across tissues often underlies phenotypic diversity^23,24^. Our study shows that the transcriptomic dynamics of coding genes between the two species across somitogenesis primarily displayed ontogeny-specific patterns, followed by species-specific ones. However, non-coding and transposable element transcripts displayed a reversed trend, suggesting higher evolutionary rates for non-coding genes.

Yet, many protein-coding genes, including those involved in the development of the NC and its derivatives, exhibited significant temporal shifts in expression trajectories between the two species. Interestingly, among these, 74 DEGs showed signatures of positive selection. Although the top DEGs with extreme interspecific sequence divergence were not directly linked to known NC-related functions, we nonetheless identified signatures of positive selection in genes involved in NC development and previously associated with NC-trait variation in cichlid fishes^30^. Overall, despite minimal interspecific morphological differences observed during somitogenesis – except for size and total numbers of somites^21^ - the variation in gene regulation and expression, especially associated with NC processes, represents an important, yet underexplored, source of species differences.

With the aim to explore how transcriptional variation connects to different modules of NC-GRN, we then focused on DEGs related to processes of NC development and differentiation of its two derivatives, namely pigmentation and craniofacial skeleton, phenotypic traits exhibiting striking diversification across cichlid radiation. Our analysis identified the *sox10* gene and its teleost-specific paralog *sox10-like* as prime candidate genes at the intersection between NC-GRN modules. The detected expression differences across species suggest that the processes of NC migration and differentiation (partly governed by *sox10*) underlie species divergence in NC-related morphologies. *in situ* HCR experiments revealed that while *sox10* paralogs are largely co-expressed in cichlid NCCs during somitogenesis, they also have distinct expression domains across several stages and anatomical structures, including expression of *sox10-like* in non-NC cells and extraembryonic tissues, possibly in line with the emergence of novel functions.

To assess functional divergence between *sox10* paralogs, we employed CRISPR/Cas9 mutagenesis system in *A. calliptera* and provided evidence for distinct roles of *sox10* and *sox10-like* in development of the NC-derived pigment and cartilage lineages, respectively. Interestingly, the observed aberrations of cartilage development in *sox10-like*-CRISPR cichlids contrast with the effects of KO mutations of its orthologues *sox10a* and *sox10b* in medaka^46^ and *sox10* in zebrafish^51^. In these teleosts, pigmentation was severely reduced but cartilage development remained unaffected (similarly to other vertebrates^52,53^). In fact, the chondrogenesis aberration seen in *sox10-like* mutants is more reminiscent of the effects of *sox9a* homozygous mutation in Nile tilapia^54^ and zebrafish^55^, as well as *sox9* in mice^56^. This suggests a different partitioning of functions among soxE family genes (specifically *sox9* and *sox10*) in cichlids compared to other teleosts. We posit that *sox10-like* acquired an essential role in chondrogenesis in the cichlid lineage, a function ancestrally performed by *sox9* in other teleosts^46,55,57^, possibly overlapping with the role of *sox9a* in cichlids^54^. In contrast, cichlid *sox10* appears to have retained its function in pigmentation development, akin to its orthologues in other teleost lineages. Additionally, severe eye malformations in *sox10-like* mutants suggest that chondrogenesis of the craniofacial skeleton might also influence ocular development. Together with observed expression differences of *prdm1a (sox10* regulator) at the anterior neural plate border, these findings implicate early variation in cranial placode development between species. Future studies will be necessary to assess the extent of functional overlap among other *soxE* family members and to delineate the roles of *sox10-like* and *sox9* paralogs in cichlid chondrogenesis, cranial placode and ocular development.

Furthermore, our comparative analysis in two divergent Malawi cichlids revealed that *sox10* duplicates show species-specific temporal and spatial variation, especially in migratory cranial NCCs. This variation could be in turn linked to pigmentation, craniofacial diversity and potentially differences in other NC-derived cell lineages, such as cranial sensory ganglia, Schwann cells and cardiomyocytes^48,58^. Together, these findings highlight the variability of the NC development in closely-related cichlids, suggesting unexplored variation in the NC gene regulatory network. A broader comparison across the whole African Great Lakes’ cichlid radiations of the genomic context of *sox10* paralogs as well as other differentially expressed genes could provide further insight into the evolution of regulatory elements driving the evolution of the NC-GRN within cichlid clade.

Functional divergence of duplicated genes is widely recognised for its role in the evolution of morphological diversity, including the expansion of the pigmentation pathway in teleosts^59^. Here, we explored the functional evolution of paralogs of *sox10*, a key factor in development of the NC across vertebrates, in the context of rapid vertebrate diversification. The uncovered functional specialisation of these two genes and variation in their expression patterns (indicating regulatory divergence) are linked to the morphological diversification of NC-derived traits during cichlid radiations and thereby contribute to the highly successful colonisation of most ecological habitats. Altogether, our findings identify NC development as major processes underlying the diversification among closely-related vertebrate species and pave groundwork for further experiments on the role of NC processes in fuelling adaptive radiation.

## METHODS

### Animal husbandry and embryo culture

Breeding stocks of *Astatotilapia calliptera* ‘Mbaka’ and *Rhamphochromis* sp. ‘chilingali’ were maintained under standardised conditions as previously described in Ref.^21^. Eggs used for RNA extractions and HCR *in situ* hybridisation experiments were collected from mouthbrooding females immediately after fertilisation and then reared individually in 1 mg/L of methylene blue (Sigma Aldrich) in water in 6-well plates (ThermoFisher Scientific) placed on an orbital shaker moving at slow speed at 27°C until needed. All experiments were conducted in compliance with the UK Home Office regulations.

### Whole embryo bulk RNA sequencing

#### Sample acquisition

For each examined species, all samples were collected from the same egg clutch. Sampling covered the entire period of somitogenesis, which coincides with NC development, with samples taken at 3-hour intervals. At each time point, at least four embryos were dissected and placed individually into either 250 μl of pre-chilled Trizol (Ambion) and stored at −80°C until RNA extraction (at least overnight) or into 1 ml of 4% PFA in 1X PBS for overnight fixation at 4°C. Embryos preserved in 4% PFA were later rinsed twice in 1X PBS and stained with 10 nM DAPI in 70% glycerol in 1X PBS overnight at 4°C, protected from light. Following a wash in 1X PBS (10 min/wash, once), the embryos were mounted on microscopy slides (ThermoFisher) with Fluoromount G (Southern Biotech) and imaged with an Olympus FV3000 confocal microscope to confirm the developmental age (somite stage) of each sampled cohort.

#### RNA extraction

All procedures were conducted on ice, unless otherwise specified. Samples stored in Trizol were thawed from −80°C. For each sample, 100 mg of 0.1 mm zirconia/silica beads (Stratech) were added before homogenization using a TissueLyser II (Qiagen) for 120 seconds at 30 Hz. The samples were then topped up to 1 ml with chilled Trizol and allowed to rest for 5 minutes. Next, 200 μL of chloroform (ThermoFisher Scientific) was added and the samples were vigorously shaken for 15 seconds, briefly vortexed and incubated at room temperature for 15 minutes. The samples were then centrifuged at 300 ×*g* for 20 minutes at 4°C. The supernatant was carefully transferred to a fresh tube and further processed using the Direct-Zol RNA Microprep Kit (Zymo) according to the manufacturer’s instructions. The quality and quantity of the extracted total RNA were assessed using Qubit (RNA HS assay, Agilent) and Tapestation (Agilent). Total RNA extracted from each embryo was submitted individually for sequencing, with quantities ranging from 135 ng to 1.3 μg per sample. All sequenced samples had eRIN values above 9.3.

#### NGS library preparation

All libraries were prepared, quality-controlled and sequenced by Novogene Corporation (China) using the Illumina NovaSeq 6000 platform to generate paired-end reads of 150 base pairs (bp). On average, 32.49 ± 2.5 Mio paired-end 150bp reads were generated per sample (Supplementary Table 1).

#### Adapter trimming and quality filtering

The adapter sequences in reads were removed, and low-quality sequences (Phred<20) were filtered out with TrimGalore (v0.6.6).

#### Mapping of RNAseq reads to reference genome

All RNAseq reads were mapped to the *Astatotilapia calliptera* genome assembly (fAstCal1.2 in Ensembl 105) using STAR v.2.7.1a^60^.

#### Gene expression quantification

The number of reads mapped to each gene in the reference genome was counted in STAR using the built-in HTSeq-count option^61^. Gene counts were normalised using the median of ratios method in DESeq2^62^ (v1.34.0). A Principal component analysis (PCA) was applied to reduce the dimensionality of the dataset using the R command prcomp (R 4.2.0).

#### Differential gene expression (DE) analysis and gene annotation

DE analysis was performed on a gene count matrix using DESeq2^62^ (v.1.34.0). Genes with mRNA counts < 10 per sample in each species were filtered out prior to analysis and technical replicates for each stage were collapsed. Heatmaps of scaled gene expression (*Z*-score per gene across all samples using mean DESeq2-normalised gene count per somite stage per species) were generated using pheatmap (v.1.0.12) and clusters of genes (unbiased complete linkage clustering) were identified and then plotted using ggplot2 (v.3.3.6).

Genes were annotated using the reference genome *A. calliptera* (fAst.cal 1.2) with biomaRt package^63^ (v.2.54.0). Significantly differentially expressed genes for each pairwise comparison were identified by filtering for log2 Fold Change above the absolute value of 0.585 (i.e. ≥ 1.5-fold difference in expression) and adjusted p-value < 0.05.

#### Gene ontology (GO) analysis

GO annotation and functional enrichment analysis were carried out using gProfiler2^64^. The full extent of the GO annotation, including the most specific GO terms for each gene or gene product, was used rather than the commonly used *GOslim* annotation, which comprises only a subset of the terms belonging to each parent domain, thus reflecting the broader biological categories. To focus the candidate search on genes involved in the NC development, a dataset comprising all DE genes with GO terms broadly associated with the developmental programme of the NC (including GO terms related to its development, migration and differentiation), development of pigmentation as well as craniofacial complex was compiled (Extended Data Table 1). These were then used to explore differences between species.

#### Transposable elements and repeat expression quantification

Transposable elements and repeats were predicted using RepeatModeler (v. 2.0.2 with LTRStruct parameter) and were then annotated using the RepeatModeler custom library in the *A. calliptera* genome using RepeatMasker (v.4.1.4; http://repeatmasker.org/). Only transposon elements (TE) were further analysed (simple repeats, tRNA, rRNA, scRNA and satellites were excluded from subsequent analyses). To quantify TE gene expression, TEcount (v.1.0.1) from the TEtranscript package^65^ was used following STAR mapping with the following parameters --chimSegmentMin 10 --winAnchorMultimapNmax 200 --outFilterMultimapNmax 100. Finally, a DESeq2-normalised gene count matrix for 1609 different transcribed TEs was generated and PCA (centred and scaled) was produced with R (prcomp).

### Population genomics

#### Detection of sites under positive selection

To identify recent signatures of positive selections, we conducted genome-wide scans to detect regions with unusually high local haplotype homozygosity and measured the extended haplotype homozygosity between the two populations (XP-EHH) using REHH (v.3.2.2)^26^. We utilised available VCF files containing biallelic SNPs from 43 samples of the Rhamphochromis genus (23.6±8.3x sequencing depth, mean±sd) and 45 randomly chosen *A. calliptera* ‘Masoko Benthic’ (16.02±1.63x) (Extended Data Figure 2a; see Methods in https://github.com/tplinderoth/cichlids/tree/master/callset and Munby, Linderoth *et al.* 2021^66^). Following the approach by Ravinet *et al.* 2018^67^, we calculated between-population extended haplotype homozygosity (xpEHH) using phased variants with a minor allele frequency (MAF) >0.05. In total, 551 significant individual sites (SNPs) were identified across all chromosomes (log[*P* value] ≥ 3), suggesting potential positive selection. Significant sites found within 50kbp (±50kbp) were grouped into ‘islands’ using bedTools (2.29.2), resulting in 154 regions containing 1 to 39 significant SNPs (3.6 SNPs on average). Genes in the closest proximity to these islands were identified using closestBed (bedTools). In total, 184 genes were linked to putative islands of selection, of which 73 were DEGs (Extended Data Table 3; 48, 5 and 20 islands located in gene bodies, promoter and intergenic regions, respectively). To identify enrichment in sites under potential positive selection within gene expression clusters, permutation tests were performed between the observed distribution of xpEHH p-values across candidate genes (25kbp regions upstream of TSS) for each gene expression cluster and the expected distribution (over chance). Expected values were calculated by randomly selecting xpEHH values (median values) across size-matched genic windows (1000x iterations; Extended Data Figure 2d).

#### Phylogenetic reconstructions

The coding sequences of *soxE* gene family members were retrieved from Ensembl (108) for inclusion in the phylogenetic analyses. Multiple sequence alignments of *soxE* gene family members were constructed using Clustal Omega^68^. Next, the alignments were trimmed with TrimAl^69^ and used to infer evolutionary relationships with the maximum likelihood method in IQ-TREE v.1.6.12^70^. In-built ModelFinder^71^ was used to infer the best-fit substitution model (TN+F+I+G4) based on the Bayesian information criterion (BIC). The branch supports for maximum likelihood analyses were obtained using the ultrafast boot-strap (UBS)^71^ with 1000 replicates. Phylogenetic tree shown in Fig. 4 was visualised using iTOL v.6^72^ and only non-significant bootstrap values are shown (≤75%).

### Genome editing

#### gRNA design and synthesis

Targets for CRISPR/Cas9 editing were selected with the CHOPCHOP software online (http://chopchop.cbu.uib.no/)^73^ using the *Astatotilapia burtoni* genome (AstBur1.0) as a reference. Sequence similarity searches with BLAST against the *A. calliptera* genome (AstCal1.2, Ensembl 108) were performed to confirm homology and test for off-target effects. Two sgRNAs targeting exon 1 were designed for *sox10* (5’-CTCGTCGTCGGATTTGACGG-3’ and 5’-CGCGGATTCCCGCGGGGAA-3’) and *sox10-like* (5’-CGGTCAGTCAGGTGCTGGACGGG-3’ and 5’-TCGTTTCCCGATCGGCATAA-3’), respectively, and purchased from Integrated DNA Technologies (ITD) as Alt-R CRISPR-Cas9 sgRNA (2 nmol).

#### Microinjection

Single-cell embryos of *Astatotilapia calliptera* ‘Mbaka’ were injected and maintained following the protocol described in Clark and Elkin *et al.* 2022^74^.

#### Embryo imaging

Injected and control embryos were imaged daily until 12 days post-fertilisation using a Leica M205 stereoscope with a DFC7000T camera under reflected light darkfield. All specimens were positioned in 1% low melting point agarose (Promega) and anaesthetised with 0.02% MS-222 (Sigma-Aldrich) if required to immobilise during imaging.

#### Genotyping

Tissue samples were taken from specimens sacrificed by overdose of 0.5% MS-222 (Sigma-Aldrich) to extract genomic DNA using PCRBIO Rapid Extract Lysis Kit (PCRBiosystems). Fragments of 190–420bp surrounding predicted deletion sites were amplified using PCRBIO HS Taq Mix Red (PCRBiosystems) with an annealing temperature of 56°C using following primer pairs: *sox10* 5’-CTGTCACCGGGTCATTCCTC-3’ and 5’-GCGTTCATTGGCCTCTTCAC-3’; *sox10-like* 5’-ATGGTCACTCACTGTCACCG-3’ and 5’-CCTCCTCGATGAATGGCCTC-3’. Amplicons were purified with QIAquick PCR Purification Kit (Qiagen) before Sanger sequencing. All protocols were conducted following manufacturer’s instructions. Sequence analysis to infer CRISPR edit sites was performed using the Synthego ICE CRISPR analysis tool (https://ice.synthego.com/).

### Cartilage preparations

Embryos were stained for cartilage following the protocol of Marconi *et al.* (2023)^21^ with following modifications: (1) all specimens were bleached to remove melanophore pigmentation using a solution of 0.05% hydrogen peroxide (Sigma) and 0.05% formamide (ThermoFisher) for 30–45 mins under light and (2) samples were cleared using first 50% then 70% glycerol:water solutions until complete sinking. Specimens were stored in 70% glycerol until imaging in 80% glycerol using a Leica M205 stereoscope with a DFC7000T camera under reflected light.

### Whole mount *in situ* hybridisation by Hybridisation Chain Reaction (HCR)

#### Reagents

The HCR probes and hairpin sets were ordered from Molecular Instruments, whereas all required buffers were made following the instructions provided by the manufacturer. The HCR probe sets (14–20 pairs per gene) were designed using target gene template sequences retrieved from *A. calliptera* genome assembly (fAstCal1.2, Ensembl 108) (Supplementary Table 3). Each probe set was designed by the manufacturer to target transcript regions common to all splicing isoforms while minimising off-target effects.

Due to a very low genetic variation in the coding sequences between study species, we used the same probe sets per each target gene for both cichlid taxa examined. Probe specificity was verified by BLAST searches against the *A. calliptera* genome available on Ensembl (AstCal 1.2) as well as against unpublished *Rhamphochromis* sp. ‘chilingali’ assembly.

### Protocol overview

Dissected embryos were fixed overnight at 4°C in 4% PFA in 1X PBS. The following day, they were rinsed twice in 1X PBST (1X PBS+0.01% Tween-20) without incubation and washed in 1X PBST (10 min/wash, twice) before a stepwise dehydration to 100% MetOH in pre-chilled solutions of 25%, 50% and 75% MetOH:PBST (10 mins/wash, at 4°C) and stored at −20°C until further analyses.

The embryos were then rehydrated from 100% MetOH to 1X PBST in reciprocal series (5 mins/wash, at 4°C), followed by washes in 1X PBST at room temperature (5 min/wash, twice).

mRNA *in situ* hybridization by chain reaction (HCR) was carried out according to the protocol of Andrews *et al.* (2020)^75^ for whole mount amphioxus embryos with the following modifications. (1) 2 pmol of each probe mixture (1 μL of 2 μM stock) per 100 μL of probe hybridization buffer were used and (2) 60 pmol of each fluorescently labelled hairpin (i.e. 2 μL of 3 μM stock) were applied per 100 μL of amplification buffer. Finally, the embryos were stained with 10nM DAPI in 70% glycerol in 1X PBS overnight at 4°C protected from light, washed in 1X SSCT (10 min/wash, twice) before mounting with Fluoromount G (Southern Biotech) on glass bottom dishes (Cellvis) without coverslip or microscopy slides (ThermoFisher Scientific) with #1.5 coverslips (Corning), depending on the size of the specimen. To prevent embryos from getting squashed when mounting on slides, thin strips of electrical tape were used as bridges to create space between the slide and a coverslip. Clear nail varnish was used to seal the edges of the slide and all samples were cured overnight at room temperature protected from light before imaging.

All *in situ* hybridization experiments were performed with multiple specimens from different clutches (at least 3 individuals per clutch, repeated at least once with specimens from alternative clutches) to fully characterise the expression patterns.

### Confocal microscopy

Imaging of dissected and stained embryos was carried out with an inverted confocal microscope Olympus FV3000 at the Imaging facility of the Department of Zoology, University of Cambridge. Since the fluorescence intensity levels were only compared as relative signals within each sample (i.e., embryo), imaging was performed using optimal laser power and emission wavelength for each sample. Sequential acquisition mode was used to minimise the signal crosstalk across channels and all images were acquired at 1024×768 resolution and 12-bit depth.

### Image processing for figure presentation

Confocal micrographs were stitched using the Olympus FV3000 software and processed with Fiji^76^ to produce optical sections, collapse z-stacks and adjust image brightness and contrast where necessary following guidelines by Schmied & Jambor (2021)^77^. The presented transverse optical sections are maximum projection across 5 adjacent slices. Auto-fluorescence and background noise in each image was removed by subtracting the average pixel intensity measured for each channel in regions of the embryo where no fluorescent signal was observed. Any remaining overexposed pixels were removed using the “Remove outliers” (radius = 0.5 pixel, threshold = 50) function in Fiji. Images presented as figures were smoothened by applying a Gaussian filter with *σ* = 0.5. Image look-up tables (LUTs) were taken from the BIOP plugin (https://imagej.net/plugins/biop-lookup-tables) and “ChrisLUTs” (Christophe Leterrier and Scott Harden; github.com/cleterrier/ChrisLUTs) package for Fiji/ImageJ. The images were processed for any background imperfections and assembled into figures in Adobe Photoshop 2023.

All plots and statistical analyses were made using R version 4.1.1. The MetBrewer package (https://github.com/BlakeRMills/MetBrewer) was used for colour palettes throughout this work.

## Supplementary Material

Supplement 1

Supplement 2

Supplement 3

Supplement 4

Supplement 5

Supplement 6

Supplement 7

Supplement 8

Supplement 9

## Data Availability

The raw RNA sequencing reads have been deposited in the Gene Expression Omnibus and will be made public upon publication. This data is made available on an open access basis for research use only. Any person who wishes to use this data for any form of commercial purpose must first enter into a commercial licensing and benefit sharing arrangement with the Government of Malawi.

## INCLUSION & ETHICS

The Malawi cichlid samples (Rhamphochromis species) were collected ethically under prescribed permits, and the results and data are published under an Access and Benefit Sharing agreement with the Government of Malawi. We acknowledge the contributions of the Malawi Department of Fisheries and the Government of Malawi for their assistance in the collection of samples and the generation of data and results.
