# Supplementary material for: Variation in Neural Crest Development and Functional Divergence of sox10 Paralogs Contribute to Morphological Diversification in Cichlid Fishes
Source: bioRxiv. 2024 Jan 31:2024.01.30.578004. Preprint. [Version 1] doi: 10.1101/2024.01.30.578004 (PMC10862805; doi:10.1101/2024.01.30.578004)
Supplement: Supplementary file 2 [file media-2.docx]

**Extended Data Table 4**

|  | ***sox9a*** | ***sox9b*** | ***sox10-like (sox10a)*** | ***sox10b*** |
| --- | --- | --- | --- | --- |
| Lake Malawi cichlids *Astatotilapia calliptera* and *Rhamphochromis* sp. 'chilingali' (this study) | / | / | Craniofacial cartilage, cardiac, ocular and pigmentation defects | Pigmentation defects |
| Nile tilapia (*Orechromis niloticus*) | Cartilage defects^1^ | / | / | / |
| medaka (*Oryzias latipes*) | / | Pigmentation defects^2^ | Pigmentation defects^3^ | Pigmentation defects^3^ |
| Zebrafish (*Danio rerio*) | Cartilage defects^4^ | / | NA | Pigmentation defects^5^ |
| Mouse (*Mus musculus*) | Skeletal malformations^6^ |  | Aberrations of cranial nerve and ganglia morphology^7^ |  |

/ - mutant phenotype unknown

NA – gene absent

1 - Li et al., 2023 *Zoological Research*

2 - Tsunogai et al., 2021 *Development, Growth & Differentiation*

3 - Nagao et al., 2018 *PLoS Genetics*

4 - Yan et al., 2005 *Development*

5 - Kelsh 2000 *BioEssays*

6 - Wagner et al., 1994 *Cell*

7 - Kuhlbrodt et al., 1998 *Journal of Neuroscience*
